# The impact of screen exposure on early literacy skills of preschool children: the mediation of parental media intervention

**DOI:** 10.3389/fpsyg.2025.1745413

**Published:** 2026-01-14

**Authors:** Jing Zu, Yixuan Zhang, Ruifang Wang

**Affiliations:** School of Preschool and Primary Education, Shenyang Normal University, Shenyang, China

**Keywords:** early literacy, parental media intervention, preschool children, screen exposure, screen media use

## Abstract

**Introduction:**

This study investigates the impact and mechanisms of screen media use on early language development in preschoolers.

**Methods:**

Via questionnaires with 516 parents of 3-6-year-olds in China, we examined children’s screen exposure and early literacy levels, then constructed a model to explore parental media intervention as a mediator.

**Results:**

Results showed: entertainment-based screen activities significantly negatively predicted early literacy, while educational use had a weak positive correlation; entertainment activities indirectly affected literacy through parental intervention, with mediating effects ranked as restrictive > educational active > caretaking active intervention.

**Discussion:**

Findings reveal how technology and family microsystems influence early literacy, offering tailored guidance for parents on screen use.

## Introduction

The research on early childhood literacy development is the focus of pedagogy, psychology, linguistics, and other disciplines. The concept of early literacy includes reading, writing, speaking, listening, and thinking skills that help children effectively use oral and written communication (Zhang et al., 2020). Research has shown that the development of literacy-related concepts, knowledge, and skills during the preschool years effectively predicts children’s future reading success (Tortorelli et al., 2022). Media ecology theory attempts to reveal the commensal relationship between human, media, and social forces in the context of ecology. In the three microsystems of the immediate home, school, and community environments, the family media environment is an important environmental factor affecting children’s growth. The presence of electronic screens everywhere in the family adds complex factors to children’s language development environment, and the family digital environment is one of the important factors promoting children’s literacy development (Papadopoulou et al., 2023). Studies have found that children are surrounded by various “screens” in the process of language learning, and the screen environment has gradually become an important environment for children to learn language within, with more and more children’s reading gradually shifting from paper to screens (Dore and Dynia, 2020). From the European “EU Kids Online” project to the United States’ “Common Sense Media” report, studies have revealed that children are using digital media at ever-younger ages, anytime and anywhere (Common Sense Media, 2020). Especially in contemporary Chinese society, the age of children’s access to the Internet tends to be younger; research shows that 56% of preschool children in China are younger than 5 years old when they first access the Internet, which indicates that children are being exposed to Internet content at very young ages (Communist Youth League Central Committee Department for Safeguarding Youth Rights and Interests, & China Internet Network Information Center, 2022). Previous studies have explored the impact of screen exposure on children’s early literacy development from two aspects: screen time and screen content. From the perspective of screen time, the duration of screen exposure has negative effects on the development of children’s vocabulary and expression ability to varying degrees and is significantly correlated with symptoms of language delay (Operto et al., 2020). Studies have found that prolonged screen media use is displacing more valuable reading activities (Twenge, 2018) and that a decrease in print reading time may be detrimental to early literacy development in children aged 3–6 years (Parish‐Morris et al., 2013). From the perspective of screen content, the impact of screen content on early childhood literacy development is more complex. Most studies have found that educational screen content has a positive impact on children’s language development, increasing vocabulary, improving word comprehension (Jing et al., 2023), promoting early literacy development, and contributing to children’s school readiness. However, non-educational television programs (entertainment or adult) have little positive impact on children’s early literacy development (Anderson and Subrahmanyam, 2017). In recent years, Phillips et al. (2008) found that, when parents create a good family reading and writing environment for children, provide high-quality screen activities, or accompany children to watch television and videos together to have parent–child interaction and guide children to learn from the screen, children attain early literacy development. This indicates that screen content may play a positive role in promoting early literacy development (Phillips et al., 2008). This suggests that parents’ media-directed behavior plays an important role in the relationship between screens and children.

Based on this, this study further explores the impact of different types of screen content on preschool children’s early literacy and the specific mechanism of parents’ media-directed behavior to provide references for parents and educators of preschool children in the use of children’s media. The first part of this paper introduces the topic of discussion, the second part contains the literature review, the third part offers the research hypothesis, the fourth part describes the research method, the fifth part shares the results and their analysis, the sixth part contains further discussion, and the seventh part presents the conclusion and suggests theoretical contributions.

## Literature review

### Children’s screen exposure

Children participate in a wide range of media forms and activities, covering multiple fields such as entertainment and learning (Valkenburg et al., 2016). Children’s screen content can be divided into two categories: educational and entertainment (Anderson and Subrahmanyam, 2017). Online classes, video calls with loved ones, or using intelligent robots can be seen as educational screen activities—think of these educational screen activities as a kind of “digital vegetable.” Conversely, content that is pure entertainment—TikTok, funny YouTube videos, and so on—can be viewed as “digital candy.” Like consuming the real thing, eating too much “digital candy” is not healthy, and children’s excessive use of entertainment screens can lead to addictions to devices such as mobile phones, resulting in a variety of negative consequences, such as obesity (Robinson et al., 2017) and myopia (Lanca and Saw, 2020). Similar to real life, “digital vegetables” seem to be healthier than “digital candy.” Some studies found that children can engage in informal digital practices while using digital media, thereby continuously improving their early literacy skills, such as word spelling and vocabulary learning (Papadopoulou et al., 2023; Jing et al., 2023). To date, the effect of educational screen activities on children’s early literacy is still controversial. On the one hand, appropriate and high-quality educational screen activities can have a positive impact on children’s early literacy (Madigan et al., 2020); on the other hand, too much screen time or access to poor-quality screen content may negatively affect children’s early literacy (Korat and Shamir, 2012). Therefore, parents and educators must consider factors such as the quality, interactivity, and adult engagement of screen activities when using them as educational tools to maximize their positive impact on early childhood literacy development. Children should watch educational videos with parental intervention because, although most children are not naturally interested in “vegetables,” “digital vegetables” may play a role in promoting their early literacy development to some extent. However, the extent of such promotion under parental intervention needs to be further elucidated.

### Early reading and writing

Clay (1966), a world-famous expert on children's language education, first put forward the idea of early literacy. She believes that the development of children's literacy begins in the early stage of a child's life and that most children acquire some degree of knowledge about language, reading, and writing before entering school. Studies have shown that there is a significant positive correlation between the development of early literacy skills and children's later reading achievements (Lonigan and Shanahan, 2009). In addition, parents reading books with preschool children has been shown to be associated with children's language growth, early literacy, and reading achievements (Hoyne and Egan, 2019). These findings support the positive effects of early literacy activities on children's language and cognitive development.İşık Uslu et al. (2022) found that the influencing factors of early literacy in preschool stage exist in two primary aspects: first are biological genetic factors, such as sex, age, temperament and so on, while, environmental factors, such as parents’ educational level, income, family parenting style, and literacy-related parenting practices and social interactions, come second. Among the environmental factors, media has gradually become an important contributor to children’s living environment and provides a reference for children’s social learning (Nechtem, 2021)). With the development of digital technology, to explain the impact of science and technology on children, Johnson and Puplampu (2008), based on the ecosystem theory (Bronfenbrenner, 1979), added a techno-subsystem as an important component outside the three microsystems (immediate home, school, and community environments). Techno-subsystems include various media platforms and devices such as television, computers, e-books, the Internet, and mobile phones. Children’s physiological, cognitive, and socio-emotional development are inseparable from their interaction with their technological environment (Johnson, 2010).

### Relationship between children’s screen exposure and early literacy

Many studies have investigated the effects of media use in early childhood, but current findings are inconsistent. Some studies suggest that children can learn from screen media and develop language skills (Barr, 2019). Some scholars have taken it to mean that media can improve children’s vocabulary (Marsh et al., 2015), and even 24-month-old children can learn from video chats or well-designed touch screens (Kirkorian et al., 2016). However, there are studies that argue the opposite: that screen media is undermining children’s language development. For example, some early childhood studies have found that very young children have difficulty picking up new words from screen media (Krcmar, 2010; Robb et al., 2009). Longitudinal studies have found that the longer the screen time of 24-month-old infants, the lower their reading volume tends to be at 36 months of age (Madigan et al., 2020). A meta-analysis of 42 studies by Madigan et al. (2020) similarly revealed that more screen time and more background noise from television were associated with poorer language skills development in children. Bhutani et al. (2023) noted that children who regularly use screens have less parent–child interaction, which can be detrimental to their development. Oktarina et al. (2024) systematically reviewed the relationship between screen time and children’s language development and found that there is a large amount of data to support the idea that children’s language development is related to screen time. Among the existing research results, there are contradictory conclusions on the relationship between screen media use and early childhood literacy, which may be because existing studies have mostly focused on the impact of screen time on preschool children’s language development while ignoring the impact of screen content on preschool children (Madigan et al., 2019; Webster et al., 2019). Existing studies have found that educational screen content has a positive impact on children’s language development, which can increase vocabulary size, improve vocabulary comprehension, promote early literacy development, and contribute to children’s school readiness (Jing et al., 2023). However, entertainment shows or adult-oriented programs have little to no positive impact on children’s early literacy development (Anderson and Subrahmanyam, 2017). Meanwhile, other studies have found that, regardless of whether screen time includes educational or entertainment screen activities, an increase in screen time has a negative impact on children’s early literacy. Children learn letters rather than language through educational media (Yuan et al., 2024; Hadley et al., 2024). The inconsistencies in the relationship between existing screen use and early reading and writing may be due to the different types of screens surveyed. Therefore, the first research question that this study focuses on is: how do different types of screen content affect preschool children’s early literacy?

Parents’ attitudes toward children’s media use and intervention behavior are also important factors affecting children’s early literacy development. DeBaryshe (1995) found that the greater the parents’ literacy level and ability, the higher the family socioeconomic status, the stronger the parents’ belief in children’s literacy development, and the better the quality of parent–child co-reading. Yeo (2015) found that parents who provide conditions for their children to read and guide them to read improve their children’s reading ability. In addition, when parents display positive emotions—for example, showing pleasure rather than scolding or disciplining the child while reading together—children show higher levels of reading ability. From the above evidence, it is clear that the way in which parents guide their children to use media and the family reading environment created for children can affect the development level of children’s early literacy. Bybee et al. (1982) first proposed that parental intervention is a multidimensional concept. In their early research on television use, parental intervention was divided into three dimensions: restrictive mediation, active mediation, and co-use. A systematic research review found that positive parental media intervention can effectively reduce children’s screen exposure time (Collier et al., 2016). In the process of children’s screen exposure, parents’ active companionship, co-viewing, and explaining media content can enhance the development of children’s critical thinking and comprehension abilities. In contrast, negative companionship, such as throwing media to children as an “electronic pacifier” or not controlling the content of children’s media use, may lead to problems like early media dependence (Yayla et al., 2025). This leads to the second research question of this study: what role does parental media intervention play in the relationship between screens and children? What are the effects of different types of parental media interventions on early language development? In summary, this study intends to study the relationship between screen exposure and early literacy of 3–6-year-old children in the critical period of language development, then analyze the mediating role of parental media intervention to further reveal the mechanism of screen exposure on the development of early childhood literacy.

## Research hypotheses

### Research hypothesis 1

As mentioned, entertainment screen activities are like digital candy. Children cannot necessarily control how much time they spend watching entertainment screen content, such as funny videos, cartoons, and advertisements, or playing video games. Children may acquire inappropriate pronunciation patterns (Alghonaim, 2020) and dialects in screen environments (Stuart-Smith et al., 2013). The displacement hypothesis suggests that, when people overuse media, there is a corresponding decrease in time spent on other activities, such as face-to-face communication. Under such a pattern, long-term screen activity will eventually replace parent–child reading and paper media use (Beck et al., 2022), which can lead to delayed early literacy development in children. Therefore, research hypothesis 1 is proposed: recreational screen activities negatively predict the early literacy ability of children aged 3–6 years.

### Research hypothesis 2

Relevant studies have found that educational screen time can promote the development of early literacy in children to a certain extent—for example, video chatting can be more helpful for children, especially infants and toddlers, in their learning (White et al., 2024; Ponti, 2023). This kind of “digital vegetable” can meet the needs of children’s language communication and conversation to a certain extent. According to media ecology theory, in the family microsystem, the impact of the family media environment on children’s early development is mainly reflected in educational screen activities; educational screen activities provide interactive learning content for children to some extent (Coyne et al., 2023), creating a certain reading and writing environment for children. Therefore, hypothesis 2 is proposed: educational screen activities promote the development of early literacy in children aged 3–6 years.

### Research hypothesis 3

**Figure 1:**
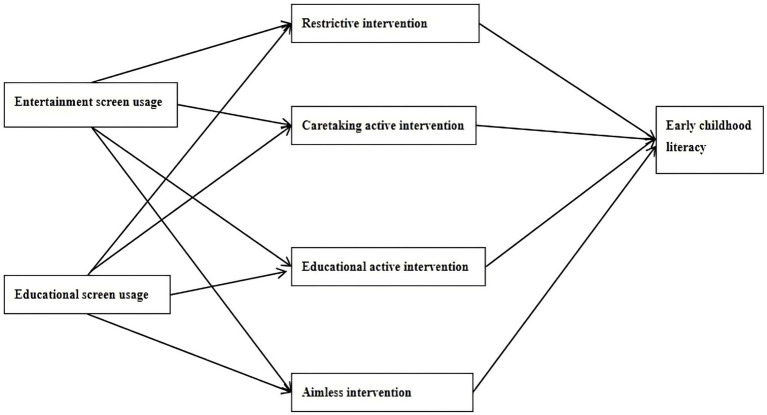
Hypothetical model diagram.

When children are exposed to screens, parents are usually more vigilant than children; some parents choose to watch screens together with their child, while some choose to restrict their child’s use, which indicates that children’s screen exposure can predict parental intervention behavior to a certain extent. Different types of parental intervention may lead to differences in early literacy development; for example, aimless parental intervention may lead to delayed development of early literacy in children, while co-viewing or media-use coaching may promote early childhood development (Przybylski and Weinstein, 2013). Media ecology theory also emphasizes the important role of parents in the family as role models for children’s language learning, to some extent driving the decision of what media children use when and where. Mahesh et al. (2024) found that interactive educational applications have a positive impact on preschool children’s language development, and this process requires the involvement of parents. Most existing studies have analyzed the impact of screen exposure or parental media intervention on early literacy in children (McArthur et al., 2021; Demir-Lira et al., 2019). As such, there is a lack of research on the mechanisms of screen exposure on early childhood literacy processes. However, parents’ effective guidance in the process of children’s media use can effectively promote children’s early literacy development (Ong’ayi et al., 2020). Therefore, referring to relevant theories and literature, research hypothesis 3 is proposed: different types of parental media intervention play a mediating role between screen exposure and early childhood literacy. The three related research hypotheses of this study are summarized, and a model diagram of the research hypotheses is put forward in Figure 1.

## Research method

### Research object

Xie et al. (2024) calculated, via a meta-analysis, an average correlation coefficient between screen exposure and early literacy of −0.112. In the present study, GPower 3.1 (Faul et al., 2009) was used to determine that the minimum sample size required for correlation analysis was 340 (*α* error probability = 0.05, test potency = 80%). Through contacting primary kindergarten teachers, online questionnaires were distributed for completion by the parents of children aged 3–6 years. Parents also signed an informed consent form to participate in the survey voluntarily. A total of 540 questionnaires were distributed, and 516 valid ones were returned, with an effective recovery rate of 95.5%. Of the 516 returned questionnaires, 60 were completed by fathers and 456 were completed by mothers. The present research opted for a combination of convenience and stratified sampling. Among the children of respondents, 103 were 3-year-olds, 167 were 4-year-olds, 138 were 5-year-olds, and 108 were 6-year-olds. In total, there were 287 boys and 229 girls, with an average age of 57.61 months. This research study was approved by the research ethics committee of Shenyang Normal University.

### Research tool

#### Children’s screen exposure

With reference to Domoff et al.'s (2019) method for calculating screen exposure time, the length of children’s use of eight types of screen media (television, gaming consoles, desktop computers, laptops, tablets, mobile phones, smart speakers with screens, and smartwatches) in a typical day was investigated, respectively. Specifically, the use time of various screen media could be divided into seven categories: ≤10 min, 11–20 min, 21–30 min, 31–60 min, 61–90 min, 91–120 min, and >120 min. Parents also recorded the type of their children’s viewing content while using the eight types of media as either educational or entertainment content, respectively. For calculation, using minutes as the unit, the midpoint of each time interval was taken, and a duration of greater than 120 min was assigned a value of 120 min. The total duration of all screen media used by children of different age groups in a day was calculated, and then the duration of each type of screen content used by children was calculated separately. This type of classification approach has been used by many scholars and is a commonly used classification method by researchers.

#### Parental media intervention

Based on the development of parent media intervention tools by Rideout (2007) and Ferreira (2017), a parent media intervention scale suitable for the Chinese cultural background was adapted. The initial questionnaire consisted of 19 questions, and four factors were obtained after factor analysis, forming four dimensions of parental media intervention, including restrictive interventions (parental restrictions on the time and content of children’s media use), caring active interventions (parents using media to care for children to facilitate their own activities, such as work, housework, etc.), educational active intervention (parents selectively using educational videos and smart toys with children), and aimless intervention (parents and children using media in a casual manner with no apparent intent). The scale contains 1–5 points to indicate responses ranging from “very inconsistent” to “very consistent.” The overall reliability coefficient of the questionnaire was 0.77, and the Cronbach’s *α* coefficients of each dimension were 0.70, 0.78, 0.86, and 0.88, respectively, which were all within the acceptable range.

#### Early literacy

The assessment of early literacy in preschool children included four aspects: textual awareness, vocabulary development, writing (drawing and doodling), and story comprehension. The details of all four aspects were indicated by parents and, due to the inclusion of complete tools for four aspects, each aspect was measured using a separate tool, as follows:

Textual awareness: Referring to the Examined in the Preschool Word and Print Awareness Measure by Justice et al. (2006), as well as the text awareness measurement tool by Shatil et al. (2000), the print awareness of preschool children was assessed using a 5-point scoring system, corresponding to questions 1–5 of the early reading and writing scale section. Statements were those such as “Children can understand the order of book pages, such as identifying the cover, previous page, next page, back cover, etc.”

Vocabulary development: The vocabulary development section referred to the vocabulary assessment questions in Morrow’s (2001) “Assessment of Children’s Language Development” scale; these were used to assess children’s vocabulary development using a 5-point scoring system corresponding to questions 6–10 on the early reading and writing scale. Statements were those such as “Children can recognize the names of themselves, familiar people, or kindergarten friends.”

Writing (painting and doodling): Sulzby (1985) defined six general writing types for children in kindergarten. Although the writing types of Chinese characters differ from the six types of writing defined by Sulzby, painting and doodling have equal effects and meanings for children of different cultures. On the basis of integrating the above reading and writing theories and listening to the suggestions of kindergarten teachers and early childhood education experts, five assessment items for writing skills in early childhood literacy were identified. We evaluated children’s writing ability using a 5-point scoring system corresponding to questions 11–15 of the early reading and writing scale. Statements were those such as “Children’s calligraphy and painting works can basically express their meanings; for example, others can recognize the numbers, symbols, objects, etc. in their works.”

Story comprehension: Restarting stories and creating or composing stories are two ancient forms of self-expression for children. Restarting a story can encourage children to use written language and make it a part of their own language. Creating stories is the primary form of storytelling; it is an advanced, comprehensive early literacy indicator that children are capable of demonstrating. Based on the comprehensive understanding theory of early childhood stories mentioned above and the advice of kindergarten teachers and early childhood education experts, eight items to assess story comprehension skills in children’s early literacy were determined (questions 16–20) and scored based on a 5-point scoring system. Statements were those such as “Children are able to adapt or create stories based on props or related contexts.”

The Cronbach’s *α* coefficient for the early literacy assessment scale was 0.912, indicating excellent internal consistency reliability (with α > 0.8 generally deemed acceptable and α > 0.9 reflecting superior reliability in educational assessments).

### Statistical processing

SPSS 25.0 software (IBM Corporation, Armonk, NY, USA) was used for data entry and statistical analysis.

## Results and analysis

### Common method bias test

Harman’s single-factor test was used for common method bias testing. Exploratory factor analysis showed that there were four factors with eigenvalues greater than 1, and the first factor explained 28.03% of the total variation, which was less than the standard of 40%. Therefore, there was no significant common method bias in this study.

### Descriptive analysis and correlation analysis

As mentioned, early media life for preschool children was divided into two categories in this study, entertainment and education, and the entertainment screen activities included watching short videos on computers/mobile phones, playing computer games, etc., while educational screen activities included watching educational videos and using intelligent robots for interaction. The present study found that the average daily time spent on entertainment screen activities by children (87.31 min/day) was greater than that spent on educational screen activities (49.54 min/day), with the most time spent watching animations on television (for an average of 31.72 min/day), as shown in Table 1.

**Table 1:** Screen exposure, early reading and writing, and parental media intervention scores and correlation (*N* = 516).

Scoring of different variables	*M ± SD*	1	2	3	4	5	6	7	8
1. Entertainment screen usage	87.31 ± 55.86	1							
2. Educational screen usage	49.54 ± 38.74	0.205**	1						
3. Average score for early reading and writing questions	3.97 ± 0.87	−0.097*	0.070	1					
4. Restrictive intervention	3.94 ± 1.13	−0.280**	0.118**	0.200**	1				
5. Caretaking proactive intervention	2.39 ± 1.00	0.194**	−0.016	−0.213**	−0.043	1			
6. Educational proactive intervention	2.85 ± 0.91	0.102*	0.307**	0.061	0.118**	0.427**	1		
7. Aimless intervention	2.15 ± 1.07	0.322**	0.017	−0.147**	−0.079	0.606**	0.398**	1	
8. Parental media intervention question average score	2.88 ± 0.66	0.093*	0.166**	−0.002	0.459**	0.741**	0.726**	0.681**	1

*p <0.05; **p <0.01; ***p <0.001.

Relevant analysis (Table 1) found that a significant correlation existed between the total score of entertainment screen activities and the total score of early reading and writing (*r* = −0.097, *p* < 0.05). However, there was no significant positive correlation between educational screen activities and early childhood reading and writing (*r* = 0.07, *p* > 0.05). Conversely, there was a significant correlation found between entertainment screen activities and various dimensions and total scores of parental media intervention, among which entertainment screen activities were significantly negatively correlated with restrictive intervention (*r* = −0.149, *p* < 0.01). There was also a significant positive correlation between educational proactive intervention, caregiving proactive intervention, and aimless intervention (*p* < 0.01). Finally, there was a significant positive correlation between entertainment screen activities and the total score of parental media intervention (*r* = 0.157, *p* < 0.01). The scores of the restrictive intervention, caregiver proactive intervention, and aimless intervention types, respectively, in parental media intervention were significantly correlated with early literacy scores, with a significant positive correlation between restrictive intervention and early literacy (*r* = 0.200, *p* < 0.01). Meanwhile, there was a significant negative correlation (*r* = −0.213 to −0. 147) between caregiving intervention, aimless intervention, and early literacy (*p* < 0.01). The results showed that entertainment screen activities may be associated with poor literacy, while positive educational screens are positively correlated with early literacy, but the correlation coefficient was not significant. In addition, relevant analysis suggests a mediating role of parental media intervention, which is reflected in the relationship between entertainment screen use and early literacy in preschool children. Restrictive intervention, educational proactive intervention, and early literacy development were positively correlated, while caregiver proactive intervention and aimless intervention were found to be negatively correlated with early literacy.

### Parallel mediation effect test of parental media intervention

**Figure 2:**
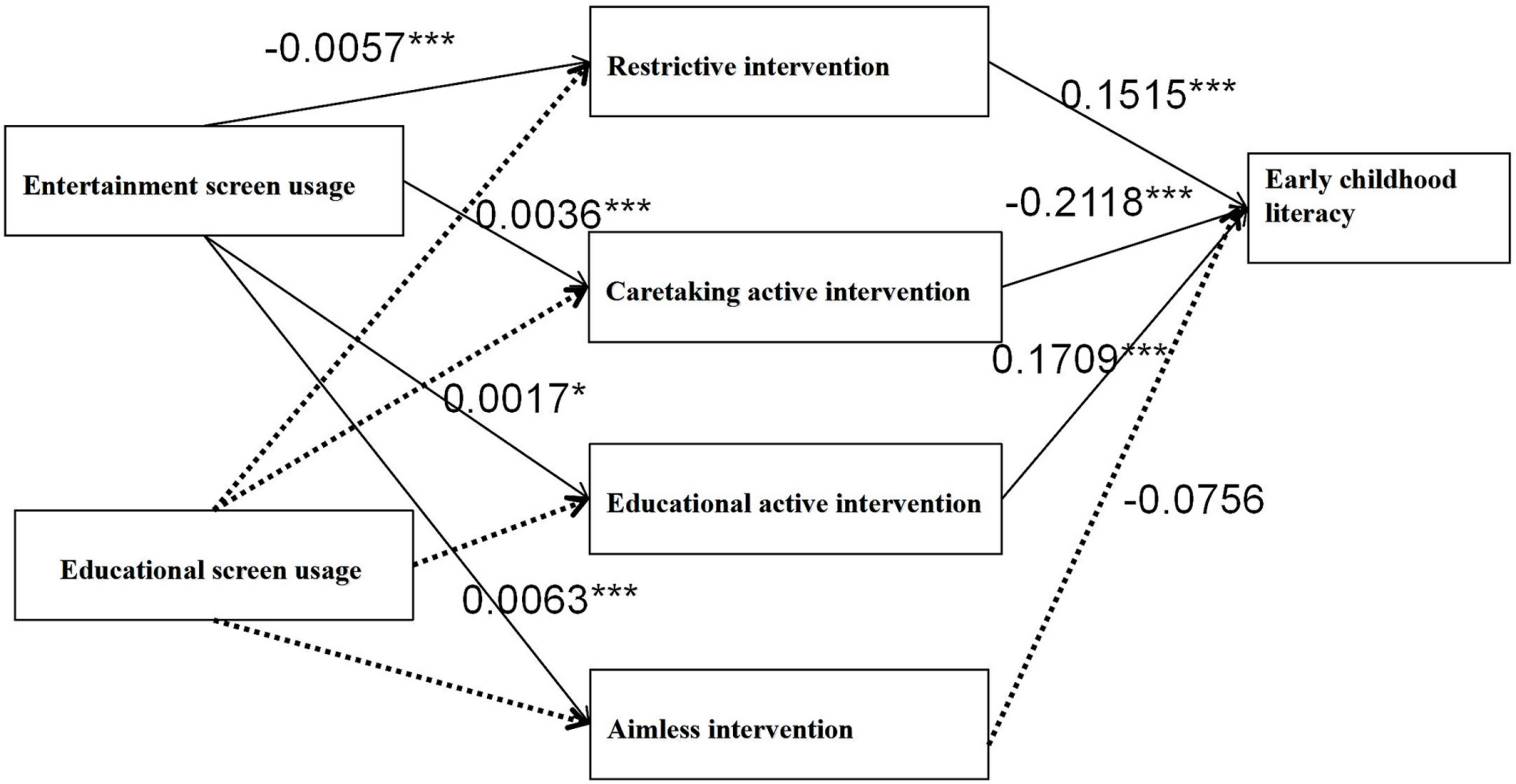
Results of model examination.

First, the mediating effect of parental media intervention on the relationship between entertainment screen activity and early literacy was tested using Model 4 (a simple mediation model) in the SPSS macro developed by Hayes (2013), while controlling for the sex and age of the children. The results showed that entertainment screen activities had a significant direct predictive effect on early childhood reading and writing abilities (*β* = −0.0024, *t* = −3.7118, *p* < 0.001), but, after intervention with mediator variables, the direct predictive effect of entertainment screen activities on early childhood reading and writing abilities was not significant (*β* = −0.0006, *t* = −1.5698, *p* > 0.05). Entertainment screen activities had a significant predictive effect on different types of parental media interventions, with a predictive effect on restrictive interventions (*β* = −0.0048, *t* = −5.8752, *p* < 0.001), a predictive effect on visual active interventions (*β* = 0.004, *t* = 5.6643, *p* < 0.001), a predictive effect on educational active interventions (*β* = 0.0019, *t* = 2.8796, *p* < 0.01), and a predictive effect on purposeless interventions (*β* = 0.0058, *t* = 7.9926, *p* < 0.001). Among the predictive effects of parental media interventions on early childhood literacy, only restrictive interventions (*β* = 0.1693, *t* = 5.7149, *p* < 0.001) and educational active interventions (*β* = 0.1464, *t* = 3.6724, *p* < 0.001) showed predictive effects, with proactive intervention for caregiving (*β* = −0.1556, *t* = −3.5737, *p* < 0.001) also observed to have a significant impact on early literacy, while the predictive effect of aimless intervention was not significant. Further analysis revealed that the comparative results of mediation showed that restrictive intervention > educational proactive intervention > caregiving intervention. There was a significant difference (*p* < 0.05) in the mediating effect between restrictive intervention and educational proactive intervention, as well as between educational proactive intervention and caregiving proactive intervention (as shown in Table 2). Assume that the model test results are shown in Figure 2.

**Table 2:** Mediating effect test of parental media intervention.

Outcome variable	Predictive variables	*β*	*t*	Boot 95% CI		*R* ^2^	*F*
				LLCI	ULCI		
Early literacy skills	Child’s sex	0.454	5.743***	0.314	0.593	0.1796	37.358***
	Preschool age	0.024	8.147***	0.018	0.030		
	Entertainment screen activities	−0.003	−3.712***	−0.004	−0.0011		
Restrictive intervention	Child’s sex	0.093	0.9298	−0.103	0.2536	0.076	14.041***
	Preschool age	−0.006	−1.9335	−0.011	0.0001		
	Entertainment screen activities	−0.005	−5.8752***	−0.0064	−0.003		
Caretaking proactive intervention	Child’s sex	−0.164	−1.9089	−0.3331	0.005	0.045	7.940***
	Preschool age	0.002	0.586	−0.0034	0.006		
	Entertainment screen activities	0.004	5.6643***	0.0026	0.0054		
Educational proactive intervention	Child’s sex	−0.133	−1.6106	−0.2955	0.029	0.020	3.416*
	Preschool age	0.001	0.1807	−0.0042	0.005		
	Entertainment screen activities	0.002	2.8796**	0.0006	0.003		
Aimless intervention	Child’s sex	−0.190	−2.136	−0.365*	−0.015	0.128	24.94***
	Preschool age	0.0042	1.6627	−0.0008	0.009		
	Entertainment screen activities	0.006	7.997***	0.004	0.007		
Early literacy skills	Child’s sex	0.405	6.148***	0.276	0.534	0.232	21.930***
	Preschool age	0.013	6.785***	0.009	0.016		
	Entertainment screen activities	−0.001	−1.570	−0.002	0.001		
	Restrictive intervention	0.169	5.715***	0.111	0.227		
	Caretaking proactive intervention	−0.156	−3.570***	−0.241	−0.070		
	Educational proactive intervention	0.146	3.672***	0.068	0.225		
	Aimless intervention	−0.038	−0.917	−0.119	0.043		

*p <0.05; **p <0.01; ***p <0.001.

In addition, the range between the upper and lower limits of the bootstrap 95% confidence interval for the mediating effect of parental media intervention did not encompass 0 (see Table 3), indicating that children’s entertainment screen activities can directly predict their early reading and writing abilities. The direct effect (−0.0009) and mediating effect (−0.0014) accounted for 39.13 and 60.87% of the total effect, respectively (see Table 3 for details). Indirect effect 1 is the use of entertainment screen activities → restrictive intervention → early reading and writing. Indirect effect 2 is the use of entertainment screen activities → educational proactive intervention → early reading and writing. Indirect effect 3 is the use of entertainment screen activities, proactive intervention for caregiving, and early reading and writing. The mediating effect of aimless intervention is not significant. Meanwhile, the mediating role of restrictive intervention is greater than that of educational proactive intervention or caring proactive intervention (as shown in Table 3).

**Table 3:** Decomposition of direct and mediating effects.

	Effect size	Boot SE	Boot 95% CI		Relative effect value
			LLCI	ULCI	
Direct	−0.0009	0.0006	−0.0021	0.0002	39.13%
Indirect	−0.0014	0.0004	−0.0021	−0.0007	60.87%
Indirect effect 1	−0.0008	0.0002	−0.0013	−0.0004	34.78%
Indirect effect 2	−0.0006	0.0002	−0.001	−0.0003	26.09%
Indirect effect 3	0.0003	0.0001	0.0001	0.0006	13.04%

## Discussion

This study provides a nuanced understanding of how different types of screen activities influence early literacy in preschool children and the critical mediating role parents play in this process. The findings illuminate the complex interplay within the family media ecology, suggesting that the type of screen content and the nature of parental intervention are pivotal in determining the impact on children’s developing literacy skills. A summary of key findings from this body of research is presented in Table A1.

### The dual nature of screen content: entertainment vs. educational

This study found that Chinese preschool children aged 3–6 years are exposed to a variety of types of screen media—mainly television, handheld gaming consoles, smartphones, smart robots, and tablets—with an average number of 4.28 tablets per family and about 136.85 min of screen time per day. Among these, watching television remains the most dominant children’s screen activity, consistent with the findings of Domingues-Montanari (2017), whose study found that watching television was the main screen activity among children aged 0–8 years. Our first key finding is the significant negative prediction of entertainment-based screen activities on early literacy, which aligns with the “displacement hypothesis” (Twenge, 2018) and a substantial body of prior research. When children spend excessive time on passive entertainment such as cartoons and short videos, it likely displaces more enriching activities like shared book reading and creative play, which are fundamental for language acquisition (Madigan et al., 2020). This is consistent with longitudinal evidence linking greater screen time to poorer language outcomes (McArthur et al., 2021). Our results reinforce the view that unguided entertainment screen time functions as a detrimental factor in the early literacy environment. Conversely, the relationship between educational screen activities and early literacy, while positive, was not statistically significant. This nuanced finding requires careful interpretation. It suggests that simply exposing children to “educational” content is insufficient for robust literacy gains. A potential explanation lies in the quality and nature of digital educational tools. As argued by Hadley et al. (2024), many literacy apps focus on narrow, isolated skills such as letter recognition (“learning letters”) rather than fostering rich, contextual language comprehension and application (“learning language”). This “drill-and-practice” approach may not effectively translate to the complex, integrated skills required for story comprehension and narrative development. Furthermore, the interactive features in educational media, if not well-designed, can become sources of distraction, potentially hindering narrative comprehension and vocabulary learning compared to traditional storybook reading (Chuang and Jamiat, 2023; Troseth et al., 2020). Therefore, the weak positive correlation we observed may reflect that some apps support basic skills, but they fall short of providing the dialogic and contextual language experiences essential for broader early literacy development.

### The central role of parental media intervention as a mediator

The most significant contribution of this study is the empirical demonstration of parental media intervention as a parallel mediator. This finding solidifies the argument that the impact of screens on children is not direct but is filtered through the family microsystem, as posited by ecological models (Johnson, 2010). The differential effects of various intervention strategies offer practical insights for parents. The finding that restrictive intervention was the strongest positive mediator is particularly insightful. In an environment saturated with enticing entertainment media, setting clear and consistent limits on screen time may be the most effective initial strategy. By restricting access to “digital candy,” parents proactively protect time and opportunity for children to engage in more valuable literacy-building activities, such as reading physical books or engaging in imaginative play (Coyne et al., 2023). This indirect support for literacy by curbing counterproductive activities appears to be highly effective, especially for younger children. In contrast, educational active intervention, which involves co-viewing and explaining content, represents a more direct, pedagogical approach. This strategy transforms screen time into an interactive, language-rich event, akin to shared reading. When parents ask questions, explain plot points, and connect on-screen content to real-life experiences, they provide the necessary scaffolding for children to extract meaning and learn new vocabulary (Strouse et al., 2018). Our results confirm that this active, guided engagement can mitigate potential negatives and leverage the educational potential of screens. The negative mediating role of Caretaking intervention (using screens as an “electronic babysitter”) underscores a significant risk. This practice is often driven by parental stress or a need for respite (Dworkin et al., 2017). However, it typically involves low-quality, non-interactive screen exposure that displaces meaningful parent–child interaction. Language development thrives on responsive, serve-and-return interactions (Romeo et al., 2018), which are absent in this scenario. Therefore, this type of intervention not only fails to support literacy but may actively contribute to delays by reducing the quantity and quality of language input children receive.

### Theoretical and practical implications

These findings extend the application of ecological systems theory to the digital age. The “techno-subsystem” is not a standalone influence; its effect is profoundly shaped by parental behaviors within the microsystem. The metaphor of “digital vegetables” and “digital candy” is useful, but our study suggests that even “vegetables” require proper “preparation”—through active parental mediation—to be truly beneficial. Practically, the results offer clear, tiered guidance for parents and educators. The primary recommendation should be to prioritize restrictive mediation to manage entertainment screen time effectively. Subsequently, efforts should focus on promoting educational active mediation, empowering parents to become active co-players and guides during their children’s educational screen use. Public health campaigns should also highlight the potential developmental opportunity costs of relying on Caretaking intervention.

### Limitations and future directions

This study has several limitations. First, its cross-sectional design precludes causal inferences. Future research should employ longitudinal or experimental designs to establish the directionality of these relationships. Second, the sample was predominantly urban; future studies should include more rural participants to enhance generalizability. Third, screen time and child outcomes were reported by parents, which may be subject to bias. Objective measures, such as digital tracking apps, would strengthen future data collection. Despite these limitations, this study clearly demonstrates that the question is not merely “how much” screen time children have, but “what” they are watching and, most importantly, “how” their parents are guiding them. Parental mediation strategies are a critical mechanism that can either attenuate the risks of screen exposure or amplify its potential benefits for early literacy development. In the future, more categorizations of screen content could be considered to dynamically examine the association between screen use and parental guidance and early childhood development. It is beneficial to develop a more fine-grained classification of screen content. While our initial categorization follows established methodologies (e.g., Domoff et al., 2019) for practical parent-reporting, we acknowledge its limitations in capturing the complexity of modern digital media. Future research is advised to incorporate “hybrid” categories (e.g., interactive e-books, educational games) and introduce dimensions such as interactivity (e.g., passive viewing, interactive gameplay, content creation) from relevant frameworks like the PACT framework to better classify screen activities. The present study revealed the bridge between screen activities and early childhood development—that is, parental media interventions—determined the existence of the mediating role of parental media interventions and suggested the role of different types of parental-mediated interventions; as such, hypothesis 3 of the study was validated. Future research could determine the distribution of participants based on the urban -rural distribution ratio of Chinese children; screen Chinese urban and rural children separately according to the corresponding ratio; and compare the duration, type, and amount of screen activities between urban and rural children to obtain more accurate data representing Chinese children’s screen exposure. In addition, future studies could use longitudinal tracking — monitoring, for example, the effects of screen exposure on preschoolers’ early literacy over time—to further expose the causal relationship between screen exposure and early literacy. Finally, future studies could install apps that record the screen activity duration on electronic devices such as smartphones and electronic tablets to help researchers collect more objective records of children’s screen media use. These methods are more accurate and objective than parental self-assessment. There may be a complex interactive causal relationship between screen exposure and parental media interventions, and the complex association between the two could be analyzed using cross-lags in the future.

## Theoretical contribution and conclusions

The present study enriches the research on the relationship between screen exposure and early literacy, providing empirical evidence for understanding the complex relationship between the two, and expands on the influence of technological microsystems in ecosystem theory on children’s early language development. The study found a juxtaposed mediating relationship between parental media intervention on screen exposure and early literacy, which could inform future guidance for parents on children’s media use, as well as a moderating effect of family socioeconomic status on the relationship between recreational screen activities and variables such as Caretaking interventions and educational interventions, which might inform future education on children’s media literacy in different types of families. Notably, the key findings are as follows. First, Chinese preschoolers in the survey sample were exposed to screen media for longer periods of time (more than 2 h per day on average), with significantly more screen time spent on entertainment content than on educational content, with an average of more than 2 h in a single day. Second, different types of screen-mediated activities had different relationships with preschoolers’ early literacy development, with recreational screen activities being significantly negatively correlated with early literacy and educational screen activities showing a non-significant positive correlation with early literacy. Finally, restrictive, caretaker-initiated, and educational-initiated interventions played a mediating role in the prediction of early literacy by recreational screen activities. A comparison of the mediating roles revealed that restrictive > educational proactive > Caretaking interventions.

## Data Availability

The raw data supporting the conclusions of this article will be made available by the authors, without undue reservation.

## Appendix

**Table A1:** Operational definitions of key constructs.

Construct	Definition and key components
Screen exposure	Activities preschool children engage in via contact with and use of screen-based electronic devices. Main categories: *entertainment-oriented* (e.g., games, cartoons) and *education-focused* (e.g., learning apps, educational videos).
Restrictive interventions	A common parental strategy in prior studies, driven by factors like limited patience or busy work schedules. Involves regulating children’s media use through rules on *screen time* (e.g., “1 hour/day”) or *content* (e.g., blocking violent shows).
Caring active interventions	Co-viewing of media (e.g., watching movies together) without purposeful discussion or interaction about the content. Limited to spontaneous emotional reactions (e.g., laughing at a funny scene) but lacks parental guidance or critical reflection on the media.
Educational active interventions	Parents actively explain media content to children (e.g., “Why is the character sharing?”) and convey educational/critical perspectives. Includes brief, intentional teaching (e.g., letter recognition in a alphabet app) during media use.
Aimless intervention	An unstructured approach where children choose any media content at any time, with no parental rules, guidance, or educational intent.
Early literacy	Pre-formal reading/writing knowledge and skills acquired by preschool children before formal schooling. Encompasses four dimensions: 1. Word awareness (e.g., recognizing print direction); 2. Vocabulary development (e.g., learning new words from books); 3. Writing (drawing/doodling as precursors to handwriting); 4. Story comprehension (e.g., retelling a story’s plot).
